# High-sensitivity C-reactive protein is associated with altered cardiac structure and function in psoriasis: The PSOCADIA study

**DOI:** 10.1016/j.ijcha.2025.101832

**Published:** 2025-11-03

**Authors:** Maria Dons, Morten Sengeløv, Kristoffer Grundtvig Skaarup, Niklas Dyrby Johansen, Mats C.H. Lassen, Sofie Bøgh-Sørensen, Julie I.H. Borchsenius, Filip Soeskov Davidovski, Nino E. Landler, Christoffer V. Nissen, Peter Riis Hansen, Brittany N. Weber, Claus Zachariae, Lone Skov, Tor Biering-Sørensen

**Affiliations:** a Cardiovascular Non-Invasive Imaging Research Laboratory, Department of Cardiology, Copenhagen University Hospital – Herlev & Gentofte, Denmark; b Center for Translational Cardiology and Pragmatic Randomized Trials, Department of Biomedical Sciences, Faculty of Health and Medical Sciences, University of Copenhagen, Denmark; c Department of Clinical Medicine, Faculty of Health and Medical Sciences, University of Copenhagen, Denmark; d Department of Dermato-Venereology, Copenhagen University Hospital – Bispebjerg & Frederiksberg, Denmark; e Division of Cardiovascular Imaging, Department of Cardiovascular Medicine, Heart and Vascular center, Brigham and Women’s Hospital, Harvard Medical School, Boston Massachusetts, USA; f Department of Dermatology and Allergy, Copenhagen University Hospital – Herlev & Gentofte, Denmark; g Copenhagen Research Group for Inflammatory Skin, Copenhagen University Hospital – Herlev & Gentofte, Denmark; h Steno Diabetes Center Copenhagen, Denmark; i Department of Cardiology, Copenhagen University Hospital – Rigshospitalet, Denmark

**Keywords:** Psoriasis, Myocardial dysfunction, Systemic inflammation, hsCRP, Prevention cardiology, Echocardiography, Cardiac imaging

## Abstract

**Background:**

High sensitivity C-reactive protein (hsCRP) is a biomarker of systemic inflammation that may be associated with cardiovascular risk in psoriasis. We assessed the relationship between hsCRP levels and cardiac structure and function in a large cross-sectional cohort study of individuals with psoriasis.

**Methods:**

Adults with psoriasis underwent hsCRP testing and transthoracic echocardiography. Myocardial dysfunction was defined as left ventricular ejection fraction < 50 % and/or global longitudinal strain (GLS) < 16 %. Diastolic dysfunction followed standard echocardiographic guidelines. Associations between hsCRP tertiles, cardiometabolic risk factors, and cardiac structure and function were evaluated. Logistic regression assessed odds of myocardial dysfunction with hsCRP > 2 mg/L.

**Results:**

972 adults with psoriasis were prospectively included (median age 54 years, 44.9 % women, 75.2 % moderate-to-severe psoriasis). Median hsCRP was 1.14 mg/L. Lower hsCRP levels were linked to greater biologic therapy use. Higher hsCRP was associated with older age, female sex, increased body mass index, and greater cardiometabolic risk factor burden.

The highest hsCRP tertile had greater rates of myocardial dysfunction (28.8 %) and diastolic dysfunction (31.3 %) compared to the lowest tertile (17.6 % and 21.8 %, respectively, p < 0.05 for both). After multivariable adjustment, increasing hsCRP was associated with impaired GLS and LVEF, and an hsCRP > 2 mg/L was independently associated with a 45 % increased odds of myocardial dysfunction (OR 1.45, 95 % CI: 1.02 – 2.07, p = 0.042).

**Conclusions:**

In psoriasis, elevated hsCRP was independently associated with impaired systolic function, reflected by reduced GLS and LVEF. These findings suggest systemic inflammation may be involved in early myocardial dysfunction in this population.

## 1 Introduction

Psoriasis has long been linked to increased cardiovascular disease (CVD) risk. This association is attributed to multiple factors, including genetic, cardiometabolic, and shared lifestyle-related factors [1].

Systemic inflammation has been identified as a key contributor to increased CVD risk in several conditions and the general population [2]. C-reactive protein (CRP) and high-sensitivity CRP (hsCRP) serve as biomarkers of systemic inflammation, with levels > 2 mg/L suggesting increased inflammatory activity [3]. However, the relationship between CRP levels, atherosclerotic CVD (ASCVD), and cardiometabolic disorders remains a chicken-or-the-egg conundrum; systemic inflammation may represent both an accelerator and consequence of these conditions [4].

Randomized trials have demonstrated that lowering hsCRP decreases vascular inflammation [5] and reduces the risk of major adverse cardiovascular events (MACE) [6]. In a UK Biobank study, CRP was associated with chronic conditions like diabetes and hypertension through body mass index (BMI) mediation. In contrast, psoriasis showed BMI-independent CRP elevation, [7] suggesting that adiposity and cardiometabolic factors alone do not fully account for the level of systemic inflammation.

This concept of residual inflammatory risk has emerged as an independent contributor to CVD, further increasing CVD risk in individuals with psoriasis beyond traditional risk factors. Notably, psoriasis has been linked to an increased risk of heart failure (HF), even in the absence of ASCVD [8]. HF is often preceded by myocardial changes detectable by echocardiography. Therefore, we investigated the association between cardiometabolic risk, systemic inflammation assessed by hsCRP, and myocardial dysfunction in a large, contemporary psoriasis cohort.

## 2 Methods

### 2.1 Study population

This is a cross-sectional analysis of a study population included in the prospective observational cohort study PSOCADIA (Prevalence and risk factors asSOciated with CArdiac comorbiDIty in psoriAsis, ClinicalTrials.gov: NCT04950218), enrolling individuals with psoriasis from January 2021 to November 2024. For future comparative studies, individuals with a diagnosis of palmoplantar pustulosis (PPP), another chronic inflammatory skin disease, were also invited to participate. Recruitment was through electronic invitation letters and dermatologists at five private practices or two out-patient clinics. Inclusion criteria were 1) age ≥18 years, 2) diagnosis of psoriasis and/or PPP, and 3) ability to provide informed consent upon participation. Exclusion criteria were 1) inability to undergo transthoracic echocardiographic assessment, 2) individuals with isolated PPP, 3) unavailable hsCRP-measurement at index study visit, and 4) hsCRP >20 mg/L to exclude individuals with ongoing viral or bacterial infection, injuries or autoimmune disease flare [9]. PSOCADIA was approved by the regional ethics board, adhered to the 1975 Declaration of Helsinki, and all participants provided written informed consent upon participation.

### 2.2 Baseline clinical information

Clinical assessment at index study visit included measurement of hip-to-waist circumference ratio, BMI, and resting blood pressure. Participants completed a detailed health questionnaire about risk factors, health status, medical therapy, physical activity level, and tobacco use. Medical history and medication were cross-referenced with data from electronic medical records and International Classification of Diseases (ICD)-10 codes.

Hypertension, diabetes, and chronic kidney disease were defined by receival of medical therapy for these conditions and/or ICD-10 codes in electronic medical records. Metabolic syndrome (MetS) was defined according to the National Cholesterol Education Program Adult Treatment Panel III (NCEP ATP III) criteria [10], requiring of ≥3 of the following risk factors; central obesity >88 cm [>35 in.] for females and >102 cm [>40 in.] for males; high-density lipoprotein (HDL) <50 mg/dL for females or <40 mg/dL for males; triglycerides >150 mg/dL or use of lipid-lowering medications; systolic blood pressure ≥130 mmHg, diastolic blood pressure ≥85 mmHg or use of antihypertensive medications; hemoglobin A1c > 5.6 %, and/or use of blood glucose-lowering medications.

ASCVD and atrial fibrillation were defined based on ICD-10 codes of condition, event, and/or prior interventional procedures or surgery for these conditions. Dyslipidemia was defined as use of lipid-lowering medical therapy and/or low-density lipoprotein (LDL) cholesterol > 3 mmol/L and/or total cholesterol > 5 mmol/L. HF was defined as having an ICD-10 code, a history of HF medical therapy, and/or HF according to echocardiography performed at index study visit. HF with preserved left ventricular ejection fraction (LVEF) (HFpEF) was defined as the presence of self-reported HF symptoms (New York Heart Association (NYHA) class II-IV dyspnea or peripheral edema), LVEF ≥50 %, elevated N-terminal pro-B-type natriuretic peptide (>125 pg/mL in sinus rhythm, >365 pg/mL in atrial fibrillation), and structural/functional cardiac abnormalities (left ventricular (LV) hypertrophy, left atrial (LA) enlargement, and/or diastolic dysfunction). HF with mid-range LVEF (HFmrEF) was defined as LVEF 41–49 % with symptoms and elevated NT-proBNP or structural/functional cardiac abnormalities. HF with reduced LVEF (HFrEF) was defined as LVEF ≤40 % with symptoms and/or medical history or ICD10 codes for HFrEF and/or medical therapy for HFrEF. HFimpEF was defined as HFrEF with LVEF ≥50 %. Valvular heart disease was defined as having ICD-10 codes for valve diseases and/or prior heart valve procedure/surgery and/or valve disease according to echocardiography performed at the index study visit.

### 2.3 High-sensitivity C-reactive protein

We collected non-fasting blood samples, which were analyzed for hsCRP (mg/L) the same day using a latex-enhanced immunoturbidimetric assay on the Atellica CH 930 system (Siemens Healthineers), with calibration using Atellica CH hsCRP CAL, verified by external quality control. The study population was stratified into tertiles based on hsCRP levels to examine associations across different hsCRP categories. HsCRP >2 mg/L was classified as indicative of low-grade systemic inflammation, as suggested by prior studies [3,6].

### 2.4 Dermatological assessment

Trained dermatologists verified diagnosis of psoriasis, and participants were assessed at baseline with Psoriasis Area and Severity Index (PASI) and nail involvement. Psoriatic arthritis (PsA) was defined as having a medical history of PsA adjudicated by a trained rheumatologist and/or having the respective ICD-10 code according to electronic medical records. All psoriasis-related characteristics and complications were adjudicated with data from the nationwide Danish Dermatology Registry, DERMBIO [11].

### 2.5 Echocardiography

Transthoracic echocardiograms were performed using GE Healthcare Vivid 9 (Horten, Norway) by experienced sonographers, following a pre-defined protocol and analyzed by a single experienced investigator blinded to clinical data with commercially available software (Viewpoint for EchoPac version 206, GE Healthcare, Horten, Norway).

LV dimensions were measured in diastole in the parasternal long-axis view at the level of the mitral valve tips. LV mass was calculated using the Devereux formula [12], and indexed with body surface area (BSA) to obtain LV mass index (LVMi). LA volume was measured using the biplane area-length method in LV end-systole and indexed with BSA to obtain LA volume index (LAVi).

Diastolic parameters included mitral valve (MV) inflow velocities (early diastolic inflow E and late diastolic inflow A) assessed by pulsed-wave (PW) Doppler in the apical four-chamber view at the level of the MV leaflet tips. PW tissue Doppler imaging was utilized to measure the peak longitudinal early diastolic myocardial velocity (e') with the sample positioned at the lateral and septal mitral annulus. E/e′ ratio was calculated by indexing E with mean e′. If identified with color Doppler, peak velocity of tricuspid regurgitation (TR) was measured using continuous wave Doppler assistance.

Diastolic dysfunction was defined according to the 2016 American Society of Echocardiography guidelines [13]. Indeterminant diastolic function according to guidelines was considered as not having diastolic dysfunction.

Systolic parameters included LVEF measured by semi-automated Simpson's biplane method in the apical four- and two-chamber view by delineating and manually adjusting the endocardial border. Two-dimensional LV speckle tracking echocardiography was conducted using the apical two-chamber, four-chamber, and apical long-axis views, dividing the LV into six segments in all three apical views. The region of interest covering the entire LV wall was traced by semi-automated software and adjusted manually by the investigator to ensure sufficient tracking. Segments with consistently poor tracking were excluded. Longitudinal strain measurements from all available segments were averaged to LV global longitudinal strain (GLS). All strain values were reported in absolute values.

Myocardial dysfunction was defined as LVEF <50 % and/or GLS <16 % [14].

### 2.6 Statistics

STATA Statistics, SE 18.0 (StataCorp, College Station, TX, USA) was used for all statistical analyses. Distribution of continuous variables was assessed using histograms and Q-Q plots. For continuous variables with a normal distribution, comparisons were made using ANOVA and data are presented as mean ± standard deviation (SD). For non-normally distributed data, the Mann-Whitney *U* test was applied and data presented as median with interquartile interval (IQI). Categorical variables were compared using Pearson’s chi-squared (χ^2^) test or Kruskal Wallis test and presented as proportions. P-for-trend was calculated using linear or logistic regression. Uni- and multivariable linear regression models were applied to assess associations between hsCRP and continuous measures of cardiac structure and function and are reported in coefficients. Associations between hsCRP and echocardiographic measures were examined in restricted cubic spline models with selection of knots based on the Akaike information criterion with the lowest value (3–7 knots tested). Due to skewness, hsCRP levels were log_2_-transformed in linear regression models to assess changes corresponding to a two-fold increase in hsCRP. Uni- and multivariable logistic regression models were used to assess associations between elevated hsCRP >2 mg/L and myocardial dysfunction. To address potential heterogeneity in pathophysiology between alterations in LVEF and GLS, we conducted sensitivity analyses for each measure. Odds ratios (ORs) and 95 % confidence intervals (95 % CIs) were displayed in Forest plots. We defined statistical significance as a p-value < 0.05.

## 3 Results

Of 2,874 adults with psoriasis and/or PPP invited to participate, 1,042 were included. After excluding individuals with isolated PPP (n = 32), those without available hsCRP (n = 27), and a hsCRP > 20 mg/L (n = 11), the study population consisted of 972 adults with psoriasis (Table 1). The median age was 54 years and the majority (75.2 %) had moderate-to-severe psoriasis. Skin disease was generally well-managed with a median PASI of 1.0, and biologics (43.2 %) and methotrexate (22.4 %) were the most used systemic psoriasis therapies. Median hsCRP was 1.13 (range: 0.17 mg/L – 18.94) mg/L.

**Table 1:** Clinical characteristics of study population stratified according to hsCRP levels.

	hsCRP ≤0.66 mg/L	0.66 < hsCRP ≤1.95 mg/L	hsCRP > 1.95 mg/L		Total		
	n = 353 (36.3 %)	n = 261 (26.9 %)	n = 358 (36.8 %)		n = 972 (100 %)	p-value	p for trend
Clinical characteristics							
Female sex, n (%)	150 (42.5)	102 (39.1)	184 (51.4)		436 (44.9)	*0.010*	*0.017*
Age, years	49.0 (35.0; 60.0)	55.0 (45.0; 64.0)	56.0 (43.0; 65.0)		54.0 (41.0; 63.0)	*<0.001*	*<0.001*
Moderate-to-severe psoriasis, n (%)	260 (73.7)	203 (77.8)	268 (74.9)		731 (75.2)	*0.50*	*0.71*
PASI	0.8 (0.0; 3.3)	0.6 (0.0; 3.0)	1.2 (0.0; 3.8)		1.0 (0.0; 3.4)	*0.07*	*0.09*
PsA, n (%)	69 (19.5)	62 (23.8)	84 (23.5)		215 (22.1)	*0.34*	*0.21*
BMI, kg/m^2^	25.2 (4.3)	27.0 (5.0)	30.2 (6.3)		27.5 (5.7)	*<0.001*	*<0.001*
Systolic BP, mmHg	130.6 (17.0)	137.7 (18.4)	139.4 (20.6)		135.7 (19.2)	*<0.001*	*<0.001*
Diastolic BP, mmHg	83.6 (10.7)	85.9 (11.8)	86.6 (12.0)		85.3 (11.6)	*<0.001*	*0.001*
Obesity (BMI > 30 kg/m^2^), n (%)	43 (12.2)	53 (20.3)	155 (43.3)		251 (25.8)	*<0.001*	*<0.001*
Waist circumference, cm	89.6 (14.2)	96.4 (15.7)	103.6 (16.6)		96.6 (16.6)	*<0.001*	*<0.001*
Waist-to-hip ratio	0.9 (0.1)	1.0 (0.1)	1.0 (0.1)		0.9 (0.1)	*<0.001*	*<0.001*
Central obesity, n (%)	94 (26.6)	112 (42.9)	247 (69.0)		453 (46.6)	*<0.001*	*<0.001*
Smoking (ever)	209 (59.2)	164 (62.8)	247 (69.0)		620 (63.8)	*0.020*	*0.007*
Physical activity weekly, n (%)	156 (57.1)	118 (54.1)	137 (44.6)		411 (51.5)	*0.010*	*0.002*

Comorbidities, n (%)							
Hypertension	67 (19.0)	80 (30.7)	135 (37.7)		282 (29.0)	*<0.001*	*<0.001*
Dyslipidemia	195 (55.2)	173 (66.3)	239 (66.8)		607 (62.4)	*<0.001*	*0.002*
Diabetes	26 (7.4)	22 (8.4)	40 (11.2)		88 (9.1)	*0.19*	*0.078*
Metabolic syndrome	77 (21.8)	90 (34.5)	181 (50.6)		348 (35.8)	*<0.001*	*<0.001*
Heart failure (any phenotype)	27 (7.6)	35 (13.4)	38 (10.6)		100 (10.3)	*0.07*	*0.20*
HFrEF	9 (2.5)	10 (3.8)	7 (2.0)		26 (2.7)	*0.35*	*0.62*
HFimpEF	3 (0.8)	2 (0.8)	2 (0.6)		7 (0.7)	*0.90*	*0.65*
HFmrEF	9 (2.5)	15 (5.7)	18 (5.0)		42 (4.3)	*0.11*	*0.11*
HFpEF	12 (3.4)	10 (3.8)	14 (3.9)		36 (3.7)	*0.93*	*0.72*
Heart valve disease	5 (1.4)	6 (2.3)	9 (2.5)		20 (2.1)	*0.56*	*0.31*
Mitral valve regurgitation	0 (−)	2 (0.8)	1 (0.3)		3 (0.3)	*0.24*	*0.52*
Mitral valve stenosis	1 (0.3)	0 (−)	1 (0.3)		2 (0.2)	*0.69*	*0.99*
Aortic valve regurgitation	2 (0.6)	2 (0.8)	3 (0.8)		7 (0.7)	*0.91*	*0.67*
Aortic valve stenosis	2 (0.6)	2 (0.8)	4 (1.1)		8 (0.8)	*0.72*	*0.42*
ASCVD	13 (3.7)	11 (4.2)	17 (4.7)		41 (4.2)	*0.78*	*0.48*
Dilated cardiomyopathy	2 (0.6)	0 (−)	0 (−)		2 (0.2)	*0.17*	*−*
Hypertrophic cardiomyopathy	1 (0.3)	0 (−)	0 (−)		1 (0.1)	*0.42*	*−*
Chronic kidney disease	6 (1.7)	2 (0.8)	12 (3.4)		20 (2.1)	*0.07*	*0.13*
Atrial fibrillation	11 (3.1)	9 (3.4)	23 (6.4)		43 (4.4)	*0.07*	*0.034*

Laboratory values							
eGFR, ml/min/1.73 m^2^	90.0 (90.0; 90.0)	90.0 (89.0; 90.0)	90.0 (88.0; 90.0)		90.0 (89.8; 90.0)	*0.19*	*0.26*
LDL cholesterol, mmol/L	2.6 (2.0; 3.2)	2.7 (2.1; 3.3)	2.8 (2.3; 3.3)		2.7 (2.1; 3.3)	*<0.001*	*0.002*
Total cholesterol, mmol/L	4.7 (4.0; 5.3)	4.8 (4.1; 5.6)	4.9 (4.3; 5.5)		4.8 (4.2; 5.5)	*0.020*	*0.018*
HsCRP, mg/L	0.36 (0.23; 0.54)	1.12 (0.88; 1.38)	3.36 (2.29; 5.72)		1.13 (0.49; 2.50)	*<0.001*	*<0.001*

Medication							
ACEi/ARB	45 (12.7)	50 (19.2)	87 (24.3)		182 (18.7)	*<0.001*	*<0.001*
Beta-blocker	5 (1.4)	11 (4.2)	7 (2.0)		23 (2.4)	*0.06*	*0.64*
SGLT2i	9 (2.5)	5 (1.9)	7 (2.0)		21 (2.2)	*0.82*	*0.59*
Psoriasis treatment regimen, n (%)						*<0.001*	*0.12*
None	27 (7.6)	18 (6.9)	39 (10.9)		84 (8.6)		
Topicals only	74 (21.0)	45 (17.2)	63 (17.6)		182 (18.7)		
Acitretin	2 (0.6)	7 (2.7)	18 (5.0)		27 (2.8)		
Roflumilast	2 (0.6)	6 (2.3)	3 (0.8)		11 (1.1)		
MTX	55 (15.6)	61 (23.4)	102 (28.5)		218 (22.4)		
Biologic	183 (51.8)	118 (45.2)	119 (33.2)		420 (43.2)		
MTX and biologic	9 (2.5)	5 (1.9)	11 (3.1)		25 (2.6)		

Biologic and small molecule therapy target, n (%)						*<0.001*	*<0.001*
TNFα	120 (62.5)	70 (56.9)	49 (37.7)		239 (53.7)		
IL-17 A/F	36 (18.8)	26 (21.1)	40 (30.8)		102 (22.9)		
IL-12/23	34 (17.7)	26 (21.1)	41 (31.5)		101 (22.7)		
JAK	1 (0.5)	0 (−)	0 (−)		1 (0.2)		
Others*	1 (0.5)	1 (0.8)	0 (−)		2 (0.4)		
* Targets: IL-6, IL-13/14							

Abbreviations: HsCRP, high sensitivity C-reactive protein; PPP, palmoplantar pustulosis; PASI, Psoriasis Area and Severity Index; PsA, psoriatic arthritis; BMI, body mass index; BP, blood pressure; HF, heart failure; HFrEF, heart failure with reduced ejection fraction; HFimpEF, heart failure with improved ejection fraction; HFmrEF, heart failure with mid-range ejection fraction; HFpEF, heart failure with preserved ejection fraction; ASCVD, atherosclerotic cardiovascular disease; eGFR, estimated glomerular filtration rate; LDL, low-density lipoprotein; ACEi, angiotensin-converting enzyme inhibitor; ARB, angiotensin II-receptor blocker; SGLT2i, sodium glucose co-transporter 2-inhibitor; MTX, methotrexate; TNFα, tumor necrosis factor alpha; IL, interleukin; JAK, Janus Kinase.

### 3.1 HsCRP and cardiometabolic risk factors

When stratified into hsCRP tertiles, individuals with the highest hsCRP levels were older and predominantly female (51.4 %, p = 0.010) (Table 1). The distribution of moderate-to-severe psoriasis and PsA did not significantly differ across hsCRP levels, and we found no difference in PASI. Nevertheless, a larger fraction received biologics in the lowest hsCRP tertile (p < 0.001).

Higher hsCRP levels were associated with higher BMI, larger WC, more prevalent central obesity, and lower levels of weekly physical activity. The prevalence of diabetes was comparable between hsCRP tertiles. However, LDL- and total cholesterol levels were slightly higher (p < 0.05 for both), and hypertension, dyslipidemia and MetS were more prevalent with increasing hsCRP (p for trend ≤0.01 for all). In total, 26 individuals (2.7 %) had HFrEF, 18 (4.3 %) HFmrEF, and 14 (3.7 %) HFpEF. Although atrial fibrillation tended to be more prevalent in the highest hsCRP tertile, the prevalences of ASCVD, valvular heart disease, and cardiomyopathies − and the distribution of HF phenotypes (HFrEF/HFmrEF/HFpEF) − did not differ across hsCRP tertiles. A higher proportion in the highest hsCRP tertile received an angiotensin-converting enzyme inhibitor (ACEi) or angiotensin II receptor blocker (ARB) (n = 87, 24.3 %; p < 0.001), primarily for hypertension (n = 85, 23.7 %; p < 0.001), whereas beta-blocker and sodium glucose-cotransporter 2-inhibitor (SGLT2i) use was comparable across tertiles.

### 3.2 HsCRP and cardiac structure and function

Measures of cardiac structure and function according to hsCRP tertiles are listed in Table 2. Compared to the low hsCRP tertile, LV wall thicknesses (interventricular septum in diastole and LV posterior wall in diastole) were slightly thicker in the higher hsCRP tertiles, but structural parameters indexed by BSA (LVMi and LAVi) were comparable between tertiles. In the higher hsCRP tertiles, diastolic function was characterized by slightly higher filling pressures (E/e′), lower septal and lateral e′ velocities, a modest increase in TR peak velocity, and a higher prevalence of overall diastolic dysfunction in the highest hsCRP tertile (31.3 % vs. 21.8 % in the lowest tertile, p = 0.020). Similarly, lower LVEF and GLS in individuals in the highest hsCRP tertile was observed, with a higher proportion exhibiting myocardial dysfunction (LVEF < 50 % or GLS < 16 %) (22.4 % vs. 17.6 % in the lowest tertile, p < 0.001).

**Table 2:** Cardiac structure, function, and echocardiographic abnormalities according to hsCRP tertiles.

Echocardiography	hsCRP ≤ 0.66 mg/L	0.66 < hsCRP ≤1.95 mg/L	hsCRP > 1.95 mg/L	Total		
	n = 353 (36.3 %)	n = 261 (26.9 %)	n = 358 (36.8 %)	n = 972 (100 %)	p-value	p for trend
Structure						
IVSd, cm	0.8 (0.2)	0.9 (0.2)	0.9 (0.2)	0.9 (0.2)	*<0.001*	*<0.001*
LVPWd, cm	0.8 (0.1)	0.8 (0.2)	0.9 (0.2)	0.8 (0.2)	*<0.001*	*0.021*
LVMi, g/m^2^	68.8 (16.5)	71.4 (16.3)	71.6 (18.2)	70.6 (17.1)	*0.06*	*0.029*
LAVi, ml/m^2^	23.0 (6.9)	23.8 (6.8)	22.8 (7.8)	23.1 (7.2)	*0.24*	*0.73*
LV systolic function						
LVEF, %	54.4 (4.5)	54.1 (4.6)	53.7 (4.4)	54.1 (4.5)	*0.13*	*0.044*
GLS, %	20.0 (3.5)	19.7 (3.6)	18.9 (4.1)	19.5 (3.8)	*<0.001*	*<0.001*

LV diastolic function						
E/e′	6.25 (5.22; 7.65)	6.86 (5.96; 8.36)	6.92 (5.62; 8.72)	6.67 (5.51; 8.25)	*<0.001*	*<0.001*
e′ lateral, cm/s	13.0 (4.0)	11.5 (3.3)	11.4 (3.6)	12.0 (3.8)	*<0.001*	*<0.001*
e' septal, cm/s	10.0 (2.8)	8.9 (2.4)	9.2 (2.5)	9.4 (2.6)	*<0.001*	*<0.001*
E/A	1.3 (0.5)	1.1 (0.4)	1.1 (0.4)	1.2 (0.4)	*<0.001*	*<0.001*
TR peak velocity, m/s	2.23 (0.30)	2.28 (0.37)	2.32 (0.34)	2.28 (0.34)	*0.020*	*0.006*
Diastolic dysfunction, n (%)	77 (21.8)	74 (28.4)	112 (31.3)	263 (27.1)	*0.020*	*0.005*
Myocardial dysfunction, n (%)	62 (17.6)	53 (20.3)	103 (28.8)	218 (22.4)	*<0.001*	*<0.001*

Abbreviations: HsCRP, high sensitivity C-reactive protein; IVSd, Interventricular septal thickness in diastole; LVPWd, Left ventricular posterior wall thickness in diastole; LVMi, Left ventricular mass index; LAVi, Left atrial volume index; LV, Left ventricular; LVEF, Left ventricular ejection fraction; GLS, Global longitudinal strain; E/e′, Early mitral inflow velocity to early diastolic mitral annular velocity ratio; e′ lateral, Early diastolic mitral annular velocity, lateral wall; e′ septal, Early diastolic mitral annular velocity, septal wall; E/A, Early (E) to late (A) ventricular filling velocities ratio; TR peak velocity, Tricuspid regurgitation peak velocity.

Higher hsCRP levels remained associated with increased LV wall thickness, as shown by positive associations with IVSd and LVPWd in the fully adjusted model (Table 3). No significant associations with hsCRP were observed for LVMi or LAVi. For diastolic function, crude associations were observed between hsCRP and altered diastolic function including higher E/e′, but these associations were attenuated after multivariable adjustment for age, sex, and obesity (Table 3). Regarding systolic function, higher hsCRP remained associated with lower LVEF (−0.31 % per doubling in hsCRP, p = 0.001), and worse GLS (−0.19 % per doubling in hsCRP, p = 0.015) after multivariable adjustment for age, sex, systemic psoriasis therapy, obesity, tobacco use, cardiometabolic risk factors, and ASCVD (Table 3).

**Table 3:** Changes in cardiac structure and function with a two-fold increase in hsCRP. Association between log_2_(hsCRP) and measures of systolic and diastolic function in uni- and multivariable linear regression models, reporting coefficients per two-fold increase in hsCRP. Model 1: Adjustment for age and sex. Model 2: Additional adjustment for psoriatic therapy. Model 3: Additional adjustment for obesity. Model 4: Additional adjustment for ACEi/ARB use. Model 5: Additional adjustment for tobacco use, hypertension, dyslipidemia, and DM, and ASCVD.

Log_2_ (hsCRP)	Crude coefficient	p-value	Model 1 coefficient	p-value	Model 2 coefficient	p-value	Model 3 coefficient	p-value	Model 4 coefficient	p-value	Model 5 coefficient	p-value
Structure												
IVSd, cm	0.02	*<0.001*	0.02	*<0.001*	0.02	*<0.001*	0.02	*<0.001*	0.01	*0.018*	0.01	*0.018*
LVPWd, cm	0.02	*<0.001*	0.02	*<0.001*	0.02	*<0.001*	0.01	*<0.001*	0.01	*0.035*	0.01	*0.030*
LVMi, g/m^2^	0.74	*0.032*	0.09	*0.09*	0.53	*0.09*	0.42	*0.18*	−0.02	*0.94*	0.07	*0.83*
LAVi, ml/m^2^	−0.04	*0.77*	−0.19	*0.18*	−0.20	*0.17*	−0.26	*0.07*	−0.24	*0.11*	−0.21	*0.16*

LV systolic function												
LVEF, %	−0.27	*0.002*	−0.33	*<0.001*	−0.34	*<0.001*	−0.32	*<0.001*	−0.30	*0.002*	−0.31	*0.001*
GLS, %	−0.35	*<0.001*	−0.37	*<0.001*	−0.38	*<0.001*	−0.37	*<0.001*	−0.18	*0.022*	−0.19	*0.015*

LV diastolic function												
E/e′	0.23	*<0.001*	0.10	*0.018*	0.10	*0.017*	0.08	*0.06*	0.03	*0.53*	0.02	*0.63*
e′ lateral, cm/s	−0.45	*<0.001*	−0.20	*0.001*	−0.20	*<0.001*	−0.20	*<0.001*	−0.12	*0.06*	−0.11	*0.07*
e' septal, cm/s	−0.26	*<0.001*	−0.10	*0.018*	−0.10	*0.018*	−0.09	*0.029*	−0.04	*0.41*	−0.03	*0.56*
E/A	−0.05	*<0.001*	−0.03	*<0.001*	−0.03	*<0.001*	−0.03	*<0.001*	−0.03	*0.002*	−0.02	*0.005*
TR peak velocity, m/s	0.03	*<0.001*	0.02	*0.024*	0.02	*0.021*	0.02	*0.024*	0.02	*0.09*	0.01	*0.0.11*

Abbreviations: HsCRP, high sensitivity C-reactive protein; IVSd, Interventricular septal thickness in diastole; LVPWd, Left ventricular posterior wall thickness in diastole; LVMi, Left ventricular mass index; LAVi, Left atrial volume index; LV, Left ventricular; LVEF, Left ventricular ejection fraction; GLS, Global longitudinal strain; E/e′, Early mitral inflow velocity to early diastolic mitral annular velocity ratio; e′ lateral, Early diastolic mitral annular velocity, lateral wall; e′ septal, Early diastolic mitral annular velocity, septal wall; E/A, Early (E) to late (A) ventricular filling velocities ratio; TR peak velocity, Tricuspid regurgitation peak velocity; ACEi, angiotensin-converting enzyme inhibitor; ARB, angiotensin II-receptor blocker, DM, diabetes mellitus; ASCVD, atherosclerotic cardiovascular disease.

**Fig. 1:**
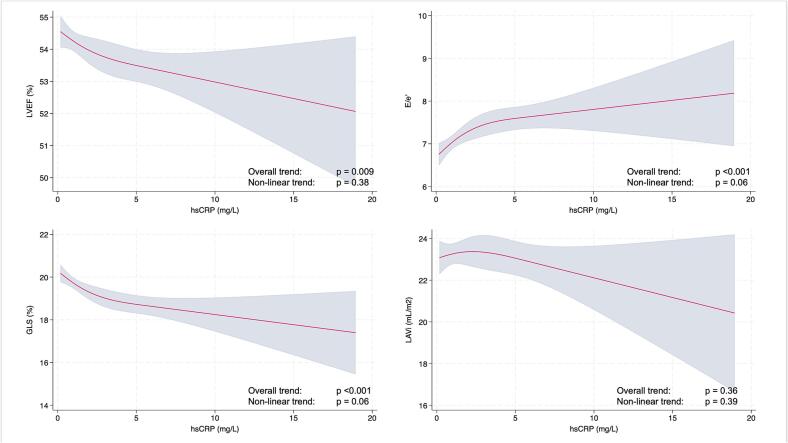
Restricted cubic spline models analyzing the association between hsCRP-levels (x-axis) and measures of systolic and diastolic function (y-axis). Red line; fitted spline regression curve, shaded area; 95% CI. HsCRP; high-sensitivity C-reactive protein; GLS, global longitudinal strain; LVEF, left ventricular ejection fraction; E/e'; ratio of early mitral inflow velocity to early diastolic mitral annular velocity; LAVi, left atrial volume index. (For interpretation of the references to colour in this figure legend, the reader is referred to the web version of this article.)

Associations between continuous hsCRP levels and selected measures of systolic and diastolic function are depicted in restricted cubic spline models (Fig. 1). Higher hsCRP levels were associated with near-linear decline in LVEF and GLS, as well as an increase in E/e′ (p < 0.05 for all) in univariable models. We found no significant association between hsCRP levels and LAVi (p = 0.36). After multivariable adjustment for age, sex, cardiometabolic risk factors, and ASCVD, increasing hsCRP remained independently associated with worse LVEF (p < 0.008) and GLS (p = 0.003).

**Fig. 2:**
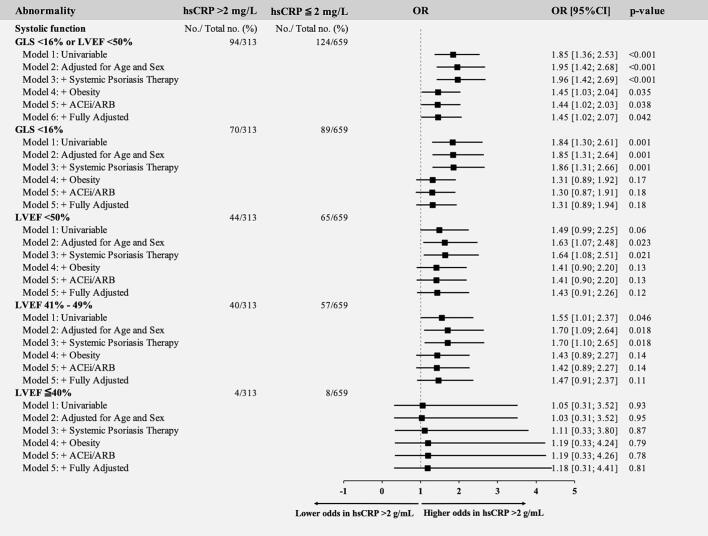

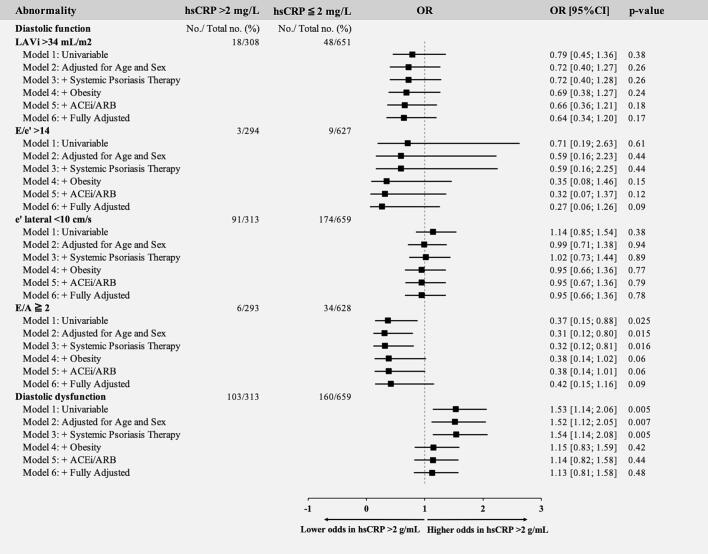
Odds ratios of systolic (A) and diastolic (B) abnormalities with a hsCRP >2 mg/L. Uni- and multivariable logistic regression models; Model 1: Unadjusted. Model 2: Adjusted for age and sex. Model 3: Adjusted for age, sex, and systemic psoriasis therapy. Model 4: Additional adjustment for obesity. Model 5: Additional adjustment for ACEi/ARB use. Model 6: Additional adjustment for tobacco use, hypertension, dyslipidemia, diabetes, and ASCVD. hsCRP, high-sensitivity C-reactive protein; ACEi, angiotensin-converting enzyme inhibitor; ARB, angiotensin II receptor blocker; ASCVD, atherosclerotic cardiovascular disease.

Among the 218 individuals exhibiting myocardial dysfunction (LVEF <50 % or GLS <16 %), 159 (72.9 %) had GLS <16 % and 109 (50.0 %) had LVEF <50 %, and 50 individuals (22.9 %) met both criteria, indicating partial but not complete overlap, with GLS identifying the larger subgroup. Assessing hsCRP >2 mg/L as a risk factor, an hsCRP >2 mg/L remained associated with 45 % increased odds of myocardial dysfunction in the fully adjusted model. In sensitivity analyses after multivariable adjustment for age, sex, and systemic psoriatic therapies, an hsCRP >2 mg/L was associated with increased odds of both diastolic dysfunction (OR 1.54, 95 % CI: 1.14; 2.08, p = 0.005), GLS <16 % (OR 1.86, 95 % CI: 1.31; 2.66, p = 0.001), LVEF <50 % (OR 1.64, 95 % CI: 1.08; 2.51, p = 0.021), and LVEF 41 %-49 % (OR 1.70, 95 % CI: 1.10; 2.65, p = 0.018) (Fig. 2). However, after additional adjustment for obesity, these associations were attenuated and no longer statistically significant (p ≥ 0.05 for all).

### 3.3 Systemic psoriatic therapies, hsCRP, and cardiac structure and function

Individuals receiving systemic psoriasis therapies such as biologics and/or methotrexate typically had moderate-to-severe inflammatory skin disease. However, those who received biologics showed hsCRP levels similar to those who received only topical therapy, of whom only 8.2 % had moderate-to-severe psoriasis (Supplemental Table 1A). Comparing systemic therapies, the subgroup receiving anti-tumor necrosis factor-alpha (TNFα) had the lowest hsCRP levels (p < 0.001) (Supplemental Table 1B). However, measures of cardiac structure and function and prevalences of myocardial dysfunction were similar across all therapies, respectively, except for lower E/A-ratio and lateral e′ in individuals receiving acitretin (Supplemental Table 2A and B).

## 4 Discussion

In this cross-sectional analysis of a large, contemporary cohort of 972 prospectively enrolled adults with psoriasis, we report several important and novel findings. First, a higher degree of systemic inflammation, assessed by hsCRP, was associated with a more adverse cardiometabolic profile and unfavorable changes in cardiac structure and function. Second, increasing hsCRP was independently associated with altered measures of systolic function. Finally, individuals with an hsCRP >2 mg/L had 48 % increased odds of myocardial dysfunction, independent of cardiometabolic risk factors.

### 4.1 Cardiometabolic risk factors and cardiac structure and function

Unsurprisingly, individuals in the highest hsCRP tertile had more adverse cardiometabolic profiles with higher BMI and more prevalent central obesity. While LVMi and LAVi were similar across hsCRP tertiles, likely due to BSA indexing [15], elevated hsCRP levels were associated with functional rather than structural abnormalities. Notably, the prevalences of ASCVD, valvular disease, and cardiomyopathies were similar across hsCRP tertiles, and HF phenotypes did not differ. Thus, the inverse associations of hsCRP with LVEF and GLS are unlikely to be explained by differential burden of established structural etiologies in this cohort.

Prior mechanistic studies suggest systemic inflammation may contribute to impaired relaxation and myocardial stiffness via obesity-related oxidative stress, insulin resistance, endothelial dysfunction, hypertensive remodeling, and neurohormonal activation [16]. Furthermore, other inflammatory pathways not captured by hsCRP have previously been associated with altered cardiac function in psoriatic disease [17]. However, interpreting hsCRP as a causal factor is complicated by its role as a marker of cumulative cardiometabolic burden [16]. In our cross-sectional study, associations between elevated hsCRP and indices of diastolic dysfunction (e.g. elevated E/e′) were evident in unadjusted models but attenuated following adjustment for key covariates, particularly obesity. A prior study found an independent association between hsCRP and E/e′ in a smaller cohort of 185 individuals with CV risk factors [18]; however, the specific covariates included in the multivariable models were not disclosed, and hsCRP was framed as a downstream marker of diastolic dysfunction, rather than a risk factor.

In contrast, elevated hsCRP > 2 mg/L remained independently associated with a 45 % increased OR of myocardial dysfunction, defined as LVEF < 50 % or GLS < 16 %, after adjusting for age, sex, systemic psoriasis therapies, ACEi/ARB use, and cardiometabolic comorbidities. Higher hsCRP levels have previously been associated with both HFpEF and HFrEF, respectively [19]. Proposed mechanisms that may underlie these associations include both direct inflammatory effects on cardiomyocytes and indirect effects through coronary microvascular dysfunction. Inflammatory cytokines such as TNFα [20] and interleukin (IL)-1 have been implicated in promoting myocyte apoptosis, fibrosis, and impaired contractility [21]. Oxidative stress and microvascular inflammation may further impair myocardial performance [22]. HsCRP may more robustly associate with reduced LVEF and GLS than with diastolic indices because it reflects systemic inflammation that directly impairs cardiomyocyte contractility through β-adrenergic desensitization, mitochondrial dysfunction, and cytokine-induced apoptosis. In contrast, diastolic dysfunction in HFpEF is often mediated though microvascular endothelial inflammation and fibrosis, mechanisms that may be more influenced by cardiometabolic inflammatory mediators [23]. For example, systolic function is more responsive to IL-1 blockade in preclinical studies with observed improvements in LVEF [24], whereas IL-1 blockade in HFpEF improved exercise tolerance, but not diastolic parameters or remodeling [25], suggesting more systemic than myocardial mechanisms.

Obesity emerged as a key confounder, markedly attenuating associations between hsCRP and both diastolic dysfunction, reduced GLS, and reduced LVEF. As a common cause of higher hsCRP and adverse myocardial mechanics, adjustment for adiposity appropriately targeted confounding and produced the largest attenuation, abolishing the dichotomous associations with most echocardiographic abnormalities, including diastolic dysfunction. This echoes findings from the ARIC study, in which accumulated hsCRP was associated with LVMi, diastolic dysfunction, and abnormal GLS in 4,011 older adults from the general population without known CVD [26]. Notably, the ARIC cohort (mean age 75 years) differed from ours (mean age 54 years), and the prevalence of immune-mediated inflammatory diseases was not reported. Nevertheless, obesity accounted for the greatest attenuation of inflammatory associations, [26] consistent with its role as both a determinant of hsCRP and an independent contributor to myocardial dysfunction [27,28]. Although a causal sequence of hsCRP increases adiposity and thereby leads to myocardial dysfunction is biologically less likely, systemic inflammation may exacerbate insulin resistance and lipotoxicity in obesity, allowing limited bidirectional reinforcement. In contrast, hypertension is best conceptualized as a mediator downstream of inflammation that contributes to myocardial dysfunction [29,30]. Consequently, additional adjustment for hypertension risks overadjustment by blocking a pathway through which systemic inflammation may act. Within this framework, our stepwise models bracket the plausible effects: base models reflect the total association between hsCRP and cardiac structure and function, obesity-adjusted models estimate a direct association net of obesity, and fully adjusted models yield a restricted direct effect that should not be interpreted as evidence against an inflammatory contribution. These distinctions help explain why hsCRP remained independently associated with lower LVEF and worse GLS after multivariable adjustment, whereas signals for diastolic indices were largely accounted for by obesity.

Given the cross-sectional design, disentangling mediation from confounding is particularly challenging, and reverse causality cannot be excluded. Longitudinal data with formal mediation analyses are needed to determine whether systemic inflammation independently contributes to myocardial dysfunction, or primarily reflects coexisting cardiometabolic risk.

### 4.2 Inflammation, myocardial dysfunction, and therapeutic targets

The distinction between the inflammatory pathways influencing systolic versus diastolic function may be important for understanding disease mechanisms and tailoring interventions [31]. Genetic data from Mendelian randomization suggest a potentially causal relationship between CRP and HF development, [32] lending biological plausibility to our observed associations between systemic inflammation and myocardial dysfunction in psoriasis. This association is further supported by growing recognition of the overlapping roles of lipid metabolism and inflammation in the pathogenesis of both atherosclerosis and HF [33]. The FOURIER (Further Cardiovascular Outcomes Research With PCSK9 Inhibitor in Patients With Elevated Risk) trial found that treatment with PCSK9-inhibitor in patients with ASCVD and LDL cholesterol ≥ 70 mg/dL despite statin therapy was linked with a greater absolute risk reduction in those with hsCRP > 3 mg/L compared to those with lower hsCRP [34], and the JUPITER (Justification for the Use of Statins in Prevention: An Intervention Trial Evaluating Rosuvastatin) trial identified individuals who benefited from intensified statin therapy by a hsCRP ≥2 mg/L in stroke prevention [35].

While traditional risk factor control remains essential, the role of targeted anti-inflammatory therapies in HF is an area of active investigation. HsCRP has been proposed as a biomarker to identify individuals who might benefit from immunomodulation therapies in HF [3]. CRP production is stimulated by IL-6 [36], and inhibitors of IL-6 are currently being investigated in HFpEF with hsCRP ≥ 2 mg/L in the Effects of Ziltivekimab Versus Placebo on Morbidity and Mortality in Patients with Heart Failure with Mildly Reduced or Preserved Ejection Fraction and Systemic Inflammation (HERMES) trial [37]. Animal models of HFpEF have demonstrated that colchicine reduces myocardial fibrosis and improves diastolic function, suggesting its potential to modulate inflammation-driven cardiac remodeling [38]. Ongoing clinical trials are currently evaluating the therapeutic impact of colchicine in HFpEF [[39], [40]]. Anti-IL-1β, inhibiting the IL-1β to IL-6 to CRP pathway, showed promising cardioprotective effects in individuals with elevated hsCRP in the Canakinumab Anti-inflammatory Thrombosis Outcome Study (CANTOS) [6], and the magnitude of hsCRP reduction in response to a single-dose of anti-IL-1β identified individuals who benefitted the most from therapy [41], supporting a lower-is-better approach.

In our psoriasis cohort, myocardial dysfunction did not differ significantly across psoriasis therapies. Although some biologic classes were associated with lower hsCRP at similar PASI, this observation should be interpreted cautiously: selection and baseline imbalances across treatment groups − including confounding by indication, whereby systemic features that prompt biologic class selection (e.g. PsA, adiposity, cardiometabolic risk) also influence hsCRP − and preferential continuation of responders could account for the pattern. Patients with more severe or systemic psoriatic disease are more likely to receive biologic therapy, and disease severity itself is linked to higher CV risk, reinforcing confounding by indication. Importantly, multivariable adjustment for treatment type did not materially change the observed associations between hsCRP and cardiac measures. Taken together, these findings should be considered hypothesis-generating and may suggest residual systemic inflammation despite adequate skin disease control. Our results should be interpreted as mechanistic: systemic inflammation relates most consistently to subclinical systolic impairment (LVEF, GLS), whereas HF phenotypes were similar across hsCRP strata. Because the cross-sectional design precludes inference about clinical HF outcomes, we plan prospective follow-up to test whether inflammatory burden and myocardial mechanics differentially predict incident HFpEF, HFmrEF, and HFrEF. Ultimately, well-powered, disease-specific trials are needed to determine whether reducing systemic inflammation, as reflected by hsCRP, mitigates myocardial dysfunction in psoriatic disease. Prior randomized controlled trials have yielded ambiguous results; [23] many were underpowered for long-term CV endpoints and reliant on surrogate biomarkers [[42], [43]].

### 4.3 HsCRP as a risk marker

We included individuals with rather well-treated psoriasis as reflected by low PASI. The observed disconnect between PASI and hsCRP underscores a clinical limitation: cutaneous disease activity, as assessed by PASI, may not fully capture systemic inflammatory burden. This accords with prior studies showing that PASI does not reliably predict circulating levels of inflammatory biomarkers of CV risk [44]. Importantly, individuals treated with biologics in our cohort had lower hsCRP, suggesting that targeted systemic therapies may attenuate systemic inflammation independent of skin clearance. Clinically, these data support joint interpretation of PASI and hsCRP for risk stratification: individuals with low PASI but persistently elevated hsCRP likely represent a discordant phenotype in whom CV risk may be underestimated by skin scores alone. In such cases, confirmation with repeat hsCRP, optimization of preventive therapy (e.g. lipid and blood pressure control), and consideration of echocardiography with GLS in the presence of symptoms or multiple risk enhancers may be warranted. Overlap analysis showed that GLS captured most cases of subclinical systolic impairment, with only ∼23 % meeting both GLS and LVEF thresholds. GLS is a sensitive marker of early myocardial dysfunction and has demonstrated strong prognostic value for MACE, even in low-risk populations [47]. Although per-doubling effects of hsCRP were modest, they were directionally consistent across models and occurred in measures with established prognostic relevance, particularly GLS. Together, this supports population-level clinical significance and the hypothesis that hsCRP may provide complementary information for CV risk management beyond cutaneous targets in psoriasis. Consistent with prevention guidelines, [33,45] hsCRP ≥2 mg/L can be treated as a risk enhancer to guide intensity of risk factor modification [46], and reassessment 8–12 weeks after therapeutic changes can document inflammatory response. Randomized controlled trials are needed to test whether a treat-to-target strategy, e.g. aiming for hsCRP <2 mg/L, would improve CV outcomes in this population, as suggested in the CANTOS trial.

### 4.4 Strengths and limitations

The strengths of our study are the prospective inclusion of a rather large and well-characterized study population of individuals with generally well-managed psoriatic disease. However, our study has several limitations. Even though participants were recruited from the whole capital region of Denmark from both specialized hospital and private dermatology clinics to capture the full spectrum of psoriatic disease severity and to enhance external validity, generalizability may be limited. Despite prospective inclusion of participants, we conducted a cross-sectional analysis, which does not allow assessment of causality. Participants will be followed up for long-term CV outcomes, allowing for longitudinal assessments of CV risk. Furthermore, medication use may introduce residual confounding. Although beta-blocker and SGLT2i use did not differ across hsCRP tertiles, and inclusion of ACEi/ARB use did not change the findings, we lacked data on treatment duration, dose, and adherence. Although Denmark has tax-funded universal health care with free access at the point of care, socioeconomic gradients in risk exposure, care-seeking, and treatment uptake persists and may contribute to residual confounding, including undiagnosed conditions such as sleep apnea. Lastly, our analysis included a single hsCRP measurement at the index study visit only, but hsCRP is reported to be relatively stable over time [48] and we excluded individuals with hsCRP levels >20 mg/L to better reflect the low-grade systemic inflammatory level.

## 5 Conclusion

In individuals with psoriasis, increasing hsCRP was associated with adverse cardiometabolic profiles and subclinical alterations in cardiac function. After multivariable adjustment, hsCRP remained independently associated with impaired systolic function, reflected by reduced GLS and LVEF, but not with diastolic dysfunction. These findings suggest that systemic inflammation, as reflected by hsCRP, is associated with early myocardial dysfunction in this population. Further research is needed to determine whether lowering hsCRP levels can influence myocardial function and reduce HF risk in psoriasis.

## CRediT authorship contribution statement

**Maria Dons:** Writing – original draft, Project administration, Methodology, Investigation, Funding acquisition, Formal analysis, Data curation. **Morten Sengeløv:** Writing – review & editing, Methodology, Investigation, Formal analysis, Data curation. **Kristoffer Grundtvig Skaarup:** . **Niklas Dyrby Johansen:** Investigation. **Mats C. H. Lassen:** Writing – review & editing, Investigation. **Sofie Bøgh-Sørensen:** Investigation. **Julie I. H. Borchsenius:** Writing – review & editing, Investigation. **Filip Soeskov Davidovski:** . **Nino E. Landler:** Writing – review & editing, Investigation. **Christoffer V. Nissen:** Writing – review & editing, Resources, Investigation. **Peter Riis Hansen:** Writing – review & editing, Resources. **Brittany N. Weber:** Writing – review & editing, Supervision, Formal analysis. **Claus Zachariae:** Writing – review & editing, Resources, Investigation, Conceptualization. **Lone Skov:** Writing – review & editing, Supervision, Resources, Project administration, Methodology, Investigation, Conceptualization. **Tor Biering-Sørensen:** Writing – review & editing, Supervision, Resources, Project administration, Methodology, Funding acquisition, Formal analysis, Conceptualization.

## Funding

The PSOCADIA study was funded by Karen Elise Jensen’s Foundation and the Lundbeck Foundation (R307-2018-3672). The funders had no role in the design or interpretation of the data.

## Declaration of competing interest

The authors declare the following financial interests/personal relationships which may be considered as potential competing interests: CVN has participated in advisory boards for UCB. CZ reports speaker and/or consultant fees for Leo Pharmaceuticals, Galderma, and UCB. LS has received research funding from Sanofi, Bristol-Myers Squibb, Almirall, Janssen Pharmaceuticals, and honoraria as consultant and/or speaker for AbbVie, Eli Lilly, Novartis, Pfizer, LEO Pharma, Janssen, Stada, Sanofi, Takeda, UCB, Almirall, Galderma, Bristol-Myers Squibb, and Sanofi. TBS has received research grants from Novartis, Pfizer, Sanofi Pasteur, GSK, Novo Nordisk, AstraZeneca, Boston Scientific and GE Healthcare, consulting fees from Novo Nordisk, IQVIA, Parexel, Amgen, CSL Seqirus, GSK and Sanofi Pasteur, and lecture fees from AstraZeneca, Bayer, Novartis, Sanofi Pasteur, GE healthcare and GSK.

## Data Availability

Data is available upon reasonable request.
