# Supplementary material for: High-sensitivity C-reactive protein is associated with altered cardiac structure and function in psoriasis: The PSOCADIA study
Source: Int J Cardiol Heart Vasc. 2025 Nov 3;61:101832. doi: 10.1016/j.ijcha.2025.101832 (PMC12630325; doi:10.1016/j.ijcha.2025.101832)
Supplement: Supplementary Data 1 [file mmc1.docx]

**Supplemental Table 1A.** HsCRP-level and psoriasis characteristics across therapies

| **Therapy** | **None** | **Topicals only** | **MTX** | **MTX + biologic** | **Biologic (any)** | **Acitretin** | **Roflumilast** | **Dimethyl fumarate** |  |
| --- | --- | --- | --- | --- | --- | --- | --- | --- | --- |
| n (%) | 84 (8.6) | 182 (18.7) | 218 (22.4) | 25 (2.6) | 420 (43.2) | 27 (2.8) | 11 (1.1) | 5 (0.5) | *p-value* |
| hsCRP, mg/L | 1.2 (0.6; 3.3) | 0.9 (0.5; 2.7) | 1.6 (0.7; 3.2) | 1.2 (0.4; 3.3) | 0.9 (0.4; 2.0) | 2.4 (1.3; 6.8) | 1.3 (0.8; 1.8) | 2.3 (0.7; 4.3) | *<0.001* |
| PASI | 3.3 (1.0; 6.4) | 3.6 (1.1; 6.0) | 1.8 (0.0; 4.0) | 0.0 (0.0; 1.8) | 0.0 (0.0; 1.8) | 1.2 (0.0; 3.3) | 2.2 (0.2; 4.3) | 3.0 (1.4; 7.3) | *<0.001* |
| PsA, n (%) | 11 (13.1) | 15 (8.2) | 38 (17.4) | 20 (80.0) | 129 (30.7) | 1 (3.7) | 0 (-) | 1 (20.0) | *<0.001* |
| Moderate-to-severe psoriasis, n (%) | 11 (13.1) | 15 (8.2) | 218 (100.0) | 25 (100.0) | 419 (99.8) | 27 (100.0) | 11 (100.0) | 5 (100.0) | *<0.001* |
|  |  |  |  |  |  |  |  |  |  |
|  |  |  |  |  |  |  |  |  |  |

**Abbreviations**: HsCRP, high-sensitivity C-reactive protein; MTX, Methotrexate; PASI, Psoriasis Area and Severity Index; PsA, Psoriatic arthritis.

**Supplemental Table 1B.** HsCRP-levels across biologics and small molecules

| **Therapy target** | **TNFα** | **IL-17 A/F** | **IL-12/23** | **Others*** |  | **All** |
| --- | --- | --- | --- | --- | --- | --- |
| n (%) | 222 (52.9) | 96 (22.9) | 100 (23.8) | 2 (0.5) | *p-value* | 420 (100.0) |
| hsCRP, mg/L | 0.7 (0.3; 1.4) | 1.3 (0.6; 2.7) | 1.3 (0.5; 2.7) | 0.9 (0.3; 1.3) | *<0.001* | 0.9 (0.4; 2.0) |
| PASI | 0.0 (0.0; 1.9) | 0.0 (0.0; 1.8) | 0.0 (0.0; 1.4) | 4.2 (-)** | *0.30* | 0.0 (0.0; 1.8) |
| PsA, n (%) | 66 (29.7) | 39 (40.6) | 23 (23.0) | 1 (50.0) | *0.05* | 129 (30.7) |
| Moderate-to-severe psoriasis, n (%) | 222 (100.0) | 96 (100.0) | 99 (99.0) | 2 (100.0) | *0.36* | 419 (99.8) |
|  |  |  |  |  |  |  |
| *Targets: JAK, IL-13/14  **missing for n = 1 |  |  |  |  |  |  |

**Abbreviations**: HsCRP, High-sensitivity C-reactive protein; TNFα, Tumor necrosis factor α; IL-17 A/F, Interleukin 17 A/F; IL-12/23, Interleukin 12/23; JAK, Janus kinase; IL-6, Interleukin 6; PASI, Psoriasis Area and Severity Index; PsA, Psoriatic arthritis; PPP, Palmoplantar pustulosis; IL-13/14, Interleukin 13/14.

**Supplemental Table 2A.** Cardiac structure, function, and prevalence of myocardial dysfunction across psoriasis therapies

| **Therapy** | **None** | **Topicals only** | **MTX** | **MTX + biologic** | **Biologic** | **Acitretin** | **Roflumilast** | **Dimethyl fumarate** |  |
| --- | --- | --- | --- | --- | --- | --- | --- | --- | --- |
| n (%) | 89 (8.9) | 193 (19.2) | 223 (22.2) | 26 (2.6) | 424 (42.3) | 32 (3.2) | 12 (1.2) | 5 (0.5) | *p-value* |
| **Structure** |  |  |  |  |  |  |  |  |  |
| IVSd, cm | 0.8 (0.2) | 0.9 (0.2) | 0.9 (0.2) | 0.9 (0.2) | 0.9 (0.2) | 1.0 (0.) | 0.9 (0.2) | 0.9 (0.2) | *0.14* |
| LVPWd, cm | 0.8 (0.2) | 0.8 (0.1) | 0.8 (0.2) | 0.9 (0.2) | 0.8 (0.2) | 0.9 (0.1) | 0.9 (0.1) | 0.9 (0.1) | *0.24* |
| LVMi, g/m2 | 69.3 (16.0) | 70.0 (15.5) | 70.2 (18.1) | 73.7 (22.1) | 70.3 (16.8) | 79.1 (20.9) | 77.1 (11.8) | 76.5 (17.6) | *0.16* |
| LAVi, ml/m^2^ | 22.2 (7.0) | 23.5 (6.9) | 23.8 (8.6) | 22.6 (5.8) | 22.8 (6.7) | 23.8 (7.0) | 25.5 (8.0) | 23.5 (7.4) | *0.51* |
| **Systolic function** |  |  |  |  |  |  |  |  |  |
| LVEF, % | 55.2 (4.1) | 54.4 (4.4) | 54.0 (5.0) | 55.0 (3.8) | 53.7 (4.4) | 54.3 (4.2) | 52.2 (4.3) | 53.8 (3.7) | *0.09* |
| GLS, % | 20.3 (3.6) | 19.5 (4.0) | 19.8 (3.7) | 18.9 (4.7) | 19.3 (3.7) | 19.0 (3.3) | 18.9 (4.0) | 18.2 (5.5) | *0.40* |
| **Diastolic function** |  |  |  |  |  |  |  |  |  |
| E/e’ | 6.80 (6.00; 8.28) | 6.22 (5.32; 8.13) | 7.00 (5.83; 8.40) | 6.38 (5.64; 7.33) | 6.46 (5.33; 8.10) | 7.24 (5.80; 10.50) | 7.30 (6.52; 9.30) | 6.76 (4.34; 12.29) | *0.012* |
| e’ lateral, cm/s | 12.10 (3.73) | 12.46 (4.09) | 11.57 (3.70) | 12.40 (3.14) | 12.08 (3.79) | 9.89 (2.82) | 10.91 (2.55) | 10.40 (2.19) | *0.020* |
| e' septal, cm/s | 9.26 (2.59) | 9.71 (2.84) | 8.99 (2.47) | 9.92 (2.50) | 9.54 (2.62) | 8.89 (2.56) | 8.73 (2.05) | 9.20 (3.27) | *0.11* |
| E/A | 1.2 (0.4) | 1.3 (0.6) | 1.1 (0.4) | 1.1 (0.4) | 1.2 (0.4) | 1.0 (0.3) | 1.1 (0.2) | 1.1 (0.5) | *0.013* |
| TR peak velocity, m/s | 2.24 (0.34) | 2.25 (0.36) | 2.28 (0.36) | 2.33 (0.33) | 2.28 (0.32) | 2.35 (0.19) | 2.29 (0.34) | 2.33 (-) | *0.94* |
| **Diastolic dysfunction**, n (%) | 17 (20.2) | 46 (25.3) | 64 (29.4) | 8 (32.0) | 117 (27.9) | 6 (22.2) | 4 (36.4) | 1 (20.0) | *0.75* |
| **Myocardial dysfunction**, n (%) | 13 (15.5) | 42 (23.1) | 53 (24.3) | 7 (28.0) | 95 (22.6) | 5 (18.5) | 2 (18.2) | 1 (20.0) | *0.82* |
|  |  |  |  |  |  |  |  |  |  |
|  |  |  |  |  |  |  |  |  |  |

**Abbreviations**: MTX, methotrexate; IVSd, Interventricular septal thickness in diastole; LVPWd, Left ventricular posterior wall thickness in diastole; LVMi, Left ventricular mass index; LAVi, Left atrial volume index; LV, Left ventricular; LVEF, Left ventricular ejection fraction; GLS, Global longitudinal strain; E/e’, Early mitral inflow velocity to early diastolic mitral annular velocity ratio; e’ lateral, Early diastolic mitral annular velocity, lateral wall; e’ septal, Early diastolic mitral annular velocity, septal wall; E/A, Early (E) to late (A) ventricular filling velocities ratio; TR peak velocity, Tricuspid regurgitation peak velocity.

**Supplemental Table 2B.** Cardiac structure, function, and prevalence of myocardial dysfunction across biologics and small molecules

| **Therapy target** | **TNFα** | **IL-17 A/F** | **IL-12/23** | **Others*** |  |
| --- | --- | --- | --- | --- | --- |
| n (%) | 222 (52.9) | 96 (22.9) | 100 (23.8) | 2 (0.5) | *p-value* |
| **Structure** |  |  |  |  |  |
| IVSd, cm | 0.9 (0.2) | 0.9 (0.2) | 0.9 (0.2) | 0.8 (0.0) | *0.85* |
| LVPWd, cm | 0.8 (0.2) | 0.8 (0.2) | 0.8 (0.2) | 0.8 (0.0) | *0.87* |
| LVMi, g/m2 | 71.5 (17.3) | 69.4 (17.3) | 68.7 (15.5) | 58.7 (6.7) | *0.35* |
| LAVi, ml/m^2^ | 23.3 (6.9) | 22.0 (5.8) | 22.4 (7.1) | 18.1 (1.6) | *0.29* |
| **Systolic function** |  |  |  |  |  |
| LVEF, % | 53.5 (4.4) | 54.0 (4.9) | 54.1 (3.6) | 49.0 (14.1) | *0.08* |
| GLS, % | 19.0 (3.9) | 19.6 (3.7) | 19.7 (3.3) | 17.0 (3.9) | *0.27* |
| **Diastolic function** |  |  |  |  |  |
| E/e’ | 6.43 (5.26; 7.85) | 6.50 (5.46; 8.41) | 6.44 (5.41; 7.97) | ** | *0.69* |
| e’ lateral, cm/s | 12.05 (3.82) | 11.80 (3.88) | 12.35 (3.66) | 14.50 (0.71) | *0.61* |
| e' septal, cm/s | 9.50 (2.65) | 9.23 (2.49) | 9.95 (2.63) | 9.00 (4.24) | *0.29* |
| E/A | 1.2 (0.5) | 1.2 (0.3) | 1.2 (0.5) | ** | *0.59* |
| TR peak velocity, m/s | 2.26 (0.30) | 2.30 (0.32) | 2.31 (0.34) | ** | *0.58* |
| **Diastolic dysfunction**, n (%) | 70 (31.5) | 23 (24.5) | 23 (23.0) | 1 (50.0) | *0.27* |
| **Myocardial dysfunction**, n (%) | 57 (25.7) | 19 (19.8) | 18 (18.0) | 1 (50.0) | *0.30* |
|  |  |  |  |  |  |
| *Targets: JAK, IL-13/14  **missing for n = 2 |  |  |  |  |  |

**Abbreviations**: TNFα, Tumor necrosis factor α; IL-17 A/F, Interleukin 17 A/F; IL-12/23, Interleukin 12/23; JAK, Janus kinase; IVSd, Interventricular septal thickness in diastole; LVPWd, Left ventricular posterior wall thickness in diastole; LVMi, Left ventricular mass index; LAVi, Left atrial volume index; LV, Left ventricular; LVEF, Left ventricular ejection fraction; GLS, Global longitudinal strain; E/e’, Early mitral inflow velocity to early diastolic mitral annular velocity ratio; e’ lateral, Early diastolic mitral annular velocity, lateral wall; e’ septal, Early diastolic mitral annular velocity, septal wall; E/A, Early (E) to late (A) ventricular filling velocities ratio; TR peak velocity, Tricuspid regurgitation peak velocity, IL-6, Interleukin 6; IL-13/14, Interleukin 13/14
